# Changes of Intestinal Oxidative Stress, Inflammation, and Gene Expression in Neonatal Diarrhoea Kids

**DOI:** 10.3389/fvets.2021.598691

**Published:** 2021-02-04

**Authors:** Yan Cheng, Chao Yang, ZhiLiang Tan, ZhiXiong He

**Affiliations:** ^1^CAS Key Laboratory for Agro-Ecological Processes in Subtropical Region, National Engineering Laboratory for Pollution Control and Waste Utilization in Livestock and Poultry Production, Hunan Provincial Key Laboratory of Animal Nutritional Physiology and Metabolic Process, Institute of Subtropical Agriculture, The Chinese Academy of Sciences, Changsha, China; ^2^Hunan Co-Innovation Center of Animal Production Safety (CICAPS), Changsha, China; ^3^University of Chinese Academy of Sciences, Beijing, China

**Keywords:** intestinal, oxidative stress, inflammation, neonatal goats, diarrhea, diagnostic biomarkers

## Abstract

Diarrhea and disorders in young goats are serious threats to the animals' health, influencing the profitability of the goat industry. There is a need to better understand the potential biomarkers that can reflect the mortality and morbidity in neonatal diarrhea goats. Ten pairs of twin kid goats from the same does (one healthy and the other diagnosed as diarrhea) with the same age under 14 days after birth were used in this study. Since gastrointestinal infection is probably the first ailment in neonatal goats, we aimed to investigate the changes in oxidative stress, inflammation, and gene expression in the gastrointestinal tract of neonatal diarrhea goats based on an epidemiological perspective. The results showed the activity of glutathione peroxidase (GSH-Px) was less (*P* < 0.05) in the jejunum in neonatal diarrhea goats compared with control goats. However, the malondialdehyde (MDA) activities in the jejunum and ileum were higher (*P* < 0.05) in neonatal diarrhea goats. There was no significant difference in the super-oxide dismutase (SOD) and catalase (CAT) activity observed between the two groups (*P* > 0.05). For the concentrations of intestinal interleukin-2 (IL2) and interleukin-6 (IL6), only the IL-2 in ileum of neonatal diarrhea goats was higher than that from healthy control goats. The transcriptomic analysis of the jejunum showed a total of 364 differential expression genes (DEGs) identified in neonatal diarrhea goats compared with control goats. The Kyoto Encyclopedia of Genes and Genomes (KEGG) functional enrichment analysis of up-regulated DEGs was mainly related to the ECM–receptor interaction and axon guidance, and the down-regulated DEGs mainly related to the Arachidonic acid metabolism, complement and coagulation cascades, and alpha-Linolenic acid metabolism. Real-time PCR results showed that the expression of most toll-like receptor-4-(TLR4) pathway-related genes and intestinal barrier function-related genes were similar in the two groups. These results suggest that neonatal diarrhea goats experienced a higher intestinal oxidative stress compared with control goats. Thus, it is possible that the antioxidant capacity of young ruminants acts as an indicator of health status and the measurements of oxidation stress may be useful as diagnostic biomarkers, reflecting the mortality and morbidity in neonatal diarrhea goats.

## Introduction

The morbidity and mortality of newborn animals in animal husbandry is relevant to animal health and welfare as well as to economic development and increased productivity. Dwyer et al. (1) have reported that the published average mortality rates of sheep in 1970–2014 from many countries and systems are stable at 15%. The overall mortality rate of lambs is often ranged from 10 to 25%, and the published estimates of goat kids mortality is between 11.5 and 37% (2). The mortality figures of newborn calves are over 30% in farms located in Tulare County, California (3). Numerous studies have clarified the causes, prevention, and treatment of neonatal disease and provided practical means (such as improving management) to reduce mortality rates. There is considerable scientific knowledge about neonatal small ruminant livestock morbidity and mortality, but it has not exerted significant effects on improving the survival. The reason may be that a substantial amount of research has been focusing on seeking and assessing solutions to the problems due to economic consideration, not on the nature of neonatal morbidity. As such, there is an urgent need to search for more effective potential biomarkers for neonatal disease diagnosis to improve animal health.

The ruminant placenta is epitheliochorial and does not allow the transfer of immune components from the mother to the young (1). Newborn goat kids are dependent on suckling for the transfer of immunoglobulin via colostrum from the does to obtain effective passive immunity. Until newborn lambs acquire passive immunity via colostrum, they are susceptible to infectious disease (4). The overall consensus is that the direct cause of newborn mortality is infectious diseases, such as neonatal diarrhea and respiratory disease, mainly caused by intestinal pathogens. It has been reported that newborn deaths are frequently caused by diarrhea due to pathogenic agents, such as *Escherichia coli*, accounting for more than 50% of the total neonatal mortality, while respiratory disorders, such as pneumonia, accounting for 15% (5). Researchers believe that enteropathogenic bacteria can influence the lungs (and vice versa) according to a theory in Chinese medicine (6). There is a close relationship between the lung and the large intestine. Thus, it has been hypothesized that a runny nose might be a typical morbidity characteristic of an intestinal pathogen infection. To date, neonatal animals classified as clinically diseased are mainly diagnosed empirically based on a range of clinical features, such as a runny nose and diarrhea. However, it is still unclear which causes will likely induce rapid clinical manifestations of neonatal diarrhea in a short time. There are few descriptions of the physiological and biochemical characteristics of newborn ruminants under pathological conditions of unknown etiology.

The intestine represents the largest component of the immune system. It contains the largest number of immune cells of any tissue in the body (more than 70% of the cells of the immune system are located in the gastrointestinal tract), and it reflects the health of young animals (7, 8). Immune cells are particularly sensitive to oxidative stress and redox has emerged as an important modality in the chemical signaling that occurs in the intestine (9). When the intracellular concentrations of reactive oxygen (ROS) are above the physiological values, it leads to oxidative stress, initiating oxidative injury and inflammatory response in gut and ultimately inducing diarrhea (10). Immune cells also could boost immunological function via regulating pro-inflammatory effector cells to reduce the secretion of pro-inflammatory cytokines, such as interleukin-2 (IL-2) and interleukin-2 (IL-6), and inhibiting pro-inflammatory pathways, such as the toll-like receptor 4 (TLR4) signaling pathway (11, 12). In this study, we hypothesized that pathogenic microorganisms induce intestinal physiological dysfunction, which leads to a disease manifested as diarrhea in newborn animals.

The transcriptome refers to all the genes in the genome transcribed in specific physiological and pathological states with a high throughput and reliable accuracy (13). It has been instrumental in the discovery of new diagnostic or therapeutic targets (14) and is widely applied to immune monitoring in inflammatory diseases to unravel pathogenic, diagnostic, and prognostic signatures (15). The objectives of this study were to evaluate the changes in the intestinal antioxidant status, inflammation state, and gene expression when neonatal goats suffered from diarrhea. We investigated the changes of the redox state and immune toxicity in diarrhea goats compared with healthy goats through determining the activities of antioxidant enzymes, such as catalase (CAT), superoxide dismutase (SOD), and glutathione peroxidase (GSH-Px); the content of malondialdehyde (MDA); secretion of IL-2 and IL-6, and immune-related genes expression by transcriptome and PCR technology. These molecular changes may shed light on the diagnosis of diarrhea based on clinical experience in neonatal goats and provide valuable clues regarding the relationship between the intestinal physiology, inflammation, and immunity.

## Materials and Methods

### Animals and Experimental Design

All of the procedures conducted in this study were in accordance with the Guide for the Animal Care and approved by the Institutional Animal Care and the Use Committee of the Institute of Subtropical Agriculture, Chinese Academy of Sciences, Changsha, China.

The experiment was carried out from March to July 2018 in a small-scale local farm based on 200 does (the Xiangdong black goat, a local meat breed) in Liuyang, Hunan Province, China. The average ambient temperature during the study was 25°C (range: 18–31°C). A group housing system consist of half-open sheds featuring with no walls on one side and the size was 20 ± 4 animals per shed. Levamisole (8 mg/kg BW) was monthly mixed into the feed for expelling parasite in the local farm. Mature goats except for pregnant ewes received additional ivermectin at its recommended dose of 0.2 mg/kg BW by subcutaneous injection to further expelling parasite in annual March and September. Goats are vaccinated (Combine Ovine/Caprine Braxy, Struck, Lamb Dysentery and Enterotoxaemia Vaccine, inactivated; Chongqing Auleon Biologicals Co., Ltd., China) in annual mid-February, except for goat kids under 2 months of age and does in late pregnancy.

In later pregnancy, does were fed twice per day at 08:30 and 17:30 with concentrate (per kg DM basis: 74.1 g whey powder, 211 g corn flour, 320 g soybean meal, 65 g fish meal, 220 g fat powder, 51 g milk powder, 8.6 g CaCO_3_, 25.3 g CaHPO_4_, 5 g NaCl, and 20 g premix) to meet the normal physiological requirements and grazed for 6 h daily. The premix (per kilogram) was composed of 119.0 g MgSO_4_·H_2_O, 2.5 g FeSO_4_·7H_2_O, 0.8 g CuSO_4_·5H_2_O, 3.0 g MnSO_4_·H_2_O, 5.0 g ZnSO_4_·H_2_O, 10.0 mg Na_2_SeO_3_, 40.0 mg KI, 30.0 mg CoCl_2_·6H_2_O, 95,000 IU vitamin A, 17,500 IU vitamin D, and 18,000 IU vitamin E. The forage was fed *ad libitum* with the same hay composed of twitch grass (dominant), ryegrass, and clover. One week before expected delivery, all pregnant dams were moved from the group housing system and housed in conventional individual pens with straw bedding located within an indoor animal facility with an average temperature of 24 ± 1°C and natural lighting. Straw beddings were replaced once a month.

The kids were ear tagged and individually reared together with their mothers in pens. The feeding trough was accessible at all times and the kids could also eat from the trough. All does and kids had free access to clean water throughout the entire experimental period. Three different investigators with extensive work experience in farm were jointly involved to judge the health of the kid goats. Health scores were evaluated by using fecal scores, nasal scores and attitude scores adapted from the University of WisThe model used for the analysis was as followsconsin calf health scoring chart (https://fyi.extension.wisc.edu/heifermgmt/files/2015/02/calf_health_scoring_chart.pdf). Nasal scores were categorized as 0: no discharge; 1: a small amount of cloudy discharge from one nostril; 2: cloudy discharge from both nostrils; and 3: excessive thick cloudy discharge from both nostrils. Fecal scores were categorized as 0: normal; 1: semi-formed, pasty; 2: loose, but stays on top of bedding; and 3: watery, sifts through bedding. Attitude scores were 0: active; 1: dull; 2: depressed; and 3: no response. Both a nasal score ≥ 1 and a fecal score ≥ 1 considered as diarrhea. And each individual aspect received a score 0 was considered as healthy.

Numerous studies have reported the highest incidence of diarrhea is the first 15 days of life (16, 17). Twin kid goats from the same does (one healthy and the other diagnosed as diarrhea) with the same age under 14 days after birth were used in this study, and a total of 10 pairs were successfully matched. The basic information of experimental kids was shown in Table 1. Once the goat kids were successful matched based on clinical experience, animals were allocated to two groups (diarrhea and healthy control experiment; *n* = 10/group) and euthanized at the same age and in a same way by intravenous overdose of sodium pentobarbital (50 mg/kg BW) and then started the slaughter test.

**Table 1:** The basic information of experimental goat kids.

**Ear tag**	**Group**	**Gender**	**Age**	**BW**	**SBW**
1C	Control	NA	3 d	NA	1.52 kg
1D	Diarrhea	NA	3 d	NA	1.36 kg
2C	Control	NA	10 d	NA	1.65 kg
2D	Diarrhea	NA	10 d	NA	1.50 kg
3C	Control	NA	10 d	NA	1.80 kg
3D	Diarrhea	NA	10 d	NA	1.50 kg
4C	Control	NA	13 d	NA	4.00 kg
4D	Diarrhea	NA	13 d	NA	2.40 kg
5C	Control	NA	5 d	NA	2.00 kg
5D	Diarrhea	NA	5 d	NA	1.75 kg
6C	Control	NA	5 d	NA	2.20 kg
6D	Diarrhea	NA	5 d	NA	1.50 kg
7C	Control	NA	8 d	NA	2.45 kg
7D	Diarrhea	NA	8 d	NA	2.65 kg
8C	Control	NA	14 d	NA	4.10 kg
8D	Diarrhea	NA	14 d	NA	2.90 kg
9C	Control	NA	7 d	NA	1.65 kg
9D	Diarrhea	NA	7 d	NA	1.85 kg
10C	Control	NA	3 d	NA	1.52 kg
10D	Diarrhea	NA	3 d	NA	1.36 kg

*C means control group; D means diarrhea group; BW means birth weight; SBW means slaughter body weight; NA means data is missing*.

### Sample Collection

Once the animals were diagnosed as diarrhea, the gut tissues (jejunum, ileum, and colon) were quickly dissected and washed three times with 0.9% sodium chloride solution. The samples were subsequently divided into three portions in an ice bath, and then immediately frozen in liquid N_2_ and stored at −80°C. One portion was used for the analyses of the oxidative index, one portion for the analyses of the changes in inflammatory factors, and the final portion was used for the analyses of the transcriptional levels of mRNA.

### Measurement of the Intestinal Oxidative Indexes and Inflammatory Cytokines

The gut tissues were homogenized (1:9 w/v) with a glass Teflon homogenizer (Potter-Elvehjem 64792-10) in a 0.9% normal saline buffer. Subsequently, the samples were centrifuged at 3,000 g for 10 min at 4°C, and the supernatant was collected to detect the activities of oxidative index and the secretion of IL-6 and IL-2 and stored at 4°C.

The activity of the SOD, CAT, GSH-Px, and MDA concentration in the gut tissues was measured by a spectrophotometric method following the instructions of the SOD, CAT, GSH-Px, and MDA detection kits, respectively (Nanjing Jiancheng Bioengineering Institute, China). The level of pro-inflammatory cytokines IL-2 and IL-6 in the gut tissues was measured by the Goat IL-2 ELISA Kit and Goat IL-6 ELISA Kit according to the manufacturer's instructions (Jiangsu Yutong Biological Technology Co., Ltd., China). All of the experiments were carried out in triplicate.

### Jejunal Transcriptome

RNA-seq analysis was carried out on the total RNA from the jejunum from neonatal goat kids in the healthy state (*n* = 4) and diseased state (*n* = 3) due to the insufficient funding. Total RNA was extracted from the jejunum tissue samples using the TRIzol reagent (Invitrogen, CA, USA). Approximate 100 mg frozen gut tissues were ground to a fine powder in liquid nitrogen using a frozen mortar and added into a 2 mL DNA/RNase free tube contained 1 mL Trizol reagent for total RNA extraction following the manufacturer's instruction. The RNA concentration and its integrity were measured using a NanoDrop 2000 (Thermo) and the RNA Nano 6000 Assay Kit of the Agilent Bioanalyzer 2100 system (Agilent Technologies, CA, USA), respectively. RNA samples, both an integrity number (RIN) >7.0 and the ratio of 28S/18S ranging from 1.8 to 2.2, were used for library construction.

A total amount of 1 μg RNA per sample was used to isolate Poly (A) mRNA with poly-T oligo attached magnetic beads (Invitrogen, CA, USA) for purification. Small pieces of the mRNA fragmentation were carried out using divalent cations under elevated temperature in NEBNext First Strand Synthesis Reaction Buffer (5 X) and were reversed to create the final cDNA library using the NEBNext UltraTM RNA Library Prep Kit (Illumina, NEB, USA) following manufacturer's recommendations. The library fragments were purified with AMPure XP system (Beckman Coulter, Beverly, USA) to select cDNA fragments of preferentially 240 bp in length and library quality was assessed on the Agilent Bioanalyzer 2100 system. Finally, the library preparations were sequenced on the Illumina NovaSeq 6000 platform (Illumina) at BioMarker Technologies and 150 bp paired-end reads were generated.

Raw data were processed by in-house perl scripts to conduct quality control testing and clean data were obtained by removing reads containing adapter, reads containing ploy-N and low-quality reads from raw data. Then RNA-Seq clean reads were mapped to the reference genome sequence (ftp://ftp.ncbi.nlm.nih.gov/genomes/all/GCF/001/704/415/GCF_001704415.1_ARS1/GCF_001704415.1_ARS1_genomic.fna.gz) using Hisat2 (18) software (https://daehwankimlab.github.io/hisat2/). The Principal Component Analysis (PCA) was conducted based on the gene expression data using R (R version 3.4.2). The expression levels of the mRNAs in each sample were estimated by fragments per kilobase of the transcript per million fragments mapped (FPKM) (19) by the following formula: FPKM = cDNA fragments/ Mapped fragments (Millions)/Transcript Length (kb). Differential expression analysis of two groups were performed to using the Bioconductor package DESeq (v 1.6.3) and the false discovery rate adjusted *P*-value (FDR) < 0.05 and |log_2_ (foldchange) | ≥ 1 was set as the threshold for significantly differential expression (20). Gene ontology (GO) enrichment analysis was performed to classify the function of differentially expressed genes (DEGs) and DEGs in categories including molecular function, biological process and cellular component using the GOseq R package based Wallenius non-central hyper-geometric distribution (21). And KOBAS (22) software were used to test the DEGs enrichment in the Kyoto Encyclopedia of Genes and Genomes (KEGG) pathways. The significant GO terms and KEGG pathways were declared at *q* < 0.05.

### Expression of Genes Related to Toll-Like Receptor 4 (TLR4) Signaling Pathway-Related Molecules and Intestinal Barrier Function

The primers of the related genes were obtained from the NCBI website, and all of the primer sequences are summarized in Supplementary Table 1. The expression of the genes related to TLR4 signaling pathway-related molecules, such as the myeloid differentiation factor 88(*MyD88*), *TLR4*, TNF receptor-associated factor-6 (*TRAF6*), interferon-beta (*IFN-*β), Interleukin-1 beta (*IL-1*β), TUMOR necrosis factor-α (*TNF-*α), *IL-6*, Leucine Rich Repeat And Pyrin Domain Containing 3 (*NLRP3*), Interferon regulatory factor 3 (*IRF3*), TANK-binding kinase-1 (*TBK1*), and Nuclear Factor kappa-Light-Chain-Enhancer of Activated B-p65 (*NF-*κ*B p65*), were analyzed by reverse transcription quantitative real-time PCR (RT-PCR).

For RT-PCR, the total RNA extraction protocol of various gut regions used in the current study was similar to what was described above. The extracted eligible RNA was reversely transcribed using PrimeScript™ RT reagent kit (RR047A, TaKaRa, Dalian, China) according to the manufacturer's instructions. Immediately after the total RNA were obtained, cDNA was produced by PrimeScript™ RT reagent kit (RR047A, TaKaRa, Dalian, China) according to the manufacturer's instructions. The reaction mixture in RT-PCR contained 0.5 μg cDNA, 5 μL SYBR Premix Ex Taq II (2×), 0.2 μL of 10 μM forward or reverse primer (Bioline, Luckenwalde, Germany) and 3.6 μL dH_2_O in a total volume of 10 μl. The relative expression of a gene was calculated relative to the mean expression of ileum in control group using the ΔΔCt method (23), with GAPDH as the internal control.

Similarly, the expressions of genes related to the intestinal barrier function, such as genes encoding tight junction (TJ) proteins, including the claudin family, such as Claudin 1 (*CLDN1*), Claudin 4 (*CLDN4*), occludin, the zonula occluden family, such as zona occludens 2 (Z*O2*), and the genes encoding mucin, such as Mucin12 (*MUC12*), Mucin13 (*MUC13*), and Mucin20 (*MUC20*), were analyzed by RT-PCR as above.

### Data Analysis

The comparison of intestinal oxidative parameters (SOD, CAT, GSH-Px, and MDA), inflammatory cytokine levels (IL2 and IL6) and relative gene expression in intestine were performed using a PROC MIXED model in SAS 9.4 (SAS Institute Inc., Cary, NC, USA), in which the health state, intestinal region and health state × intestinal region were used as the fixed effect, while the neonatal goat was the random effect. Individual animal was the experimental unit, and Tukey's test was used to compare least squares means. The model used for the analysis was as follows:

$$\begin{array}{l} \text{Y}=\mu +\text{Ti}+\text{Rj}+\text{TRij}+\text{Gik}+\text{εijk} \end{array}$$

Where *Y* is the dependent variable, μ is the population mean for the variable, *Ti* is health state (*i* = healthy, diarrhea) as the fixed effect, *Rj* is intestinal region (*j* = jejunum, ileum, and colon) as the fixed effect, *TRij* is the interaction between health state and intestinal region, *Gik* is kid goat (*k* = 1,2,3,…10) as the random effect and ε*ijk* is the random error associated with the observation of *ijk*.

The level for significance was set at < 0.05, and all results were expressed as mean ± standard error of the mean.

## Results

### Activities of the Antioxidant Enzymes and Oxidative Products in Gut Tissues

**Figure 1:**
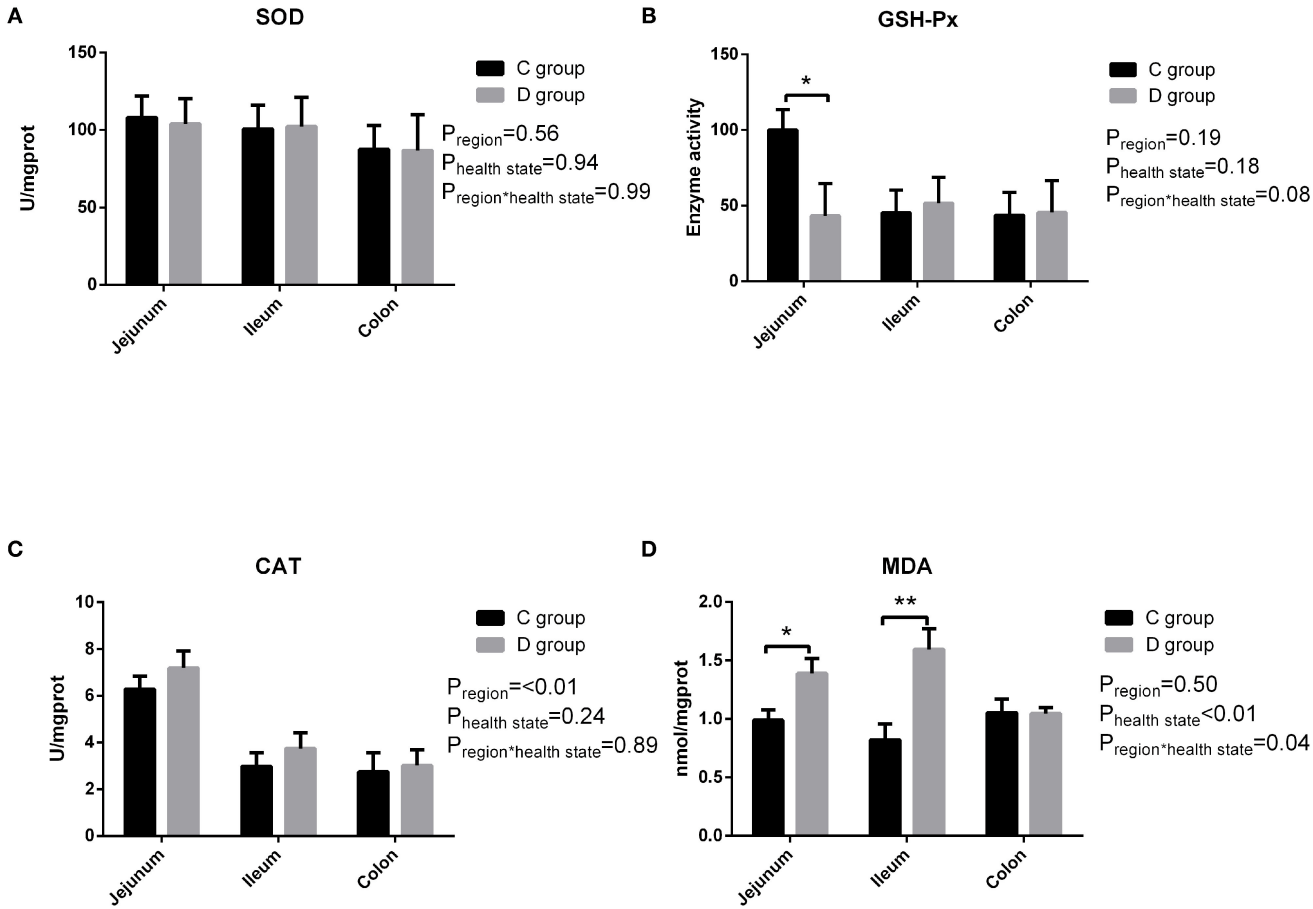
The changes of intestinal SOD **(A)**, GSH-Px **(B)**, CAT **(C)**, and MDA **(D)** in neonatal diarrhea goats. C group means control group; D group means diarrhea group. Results are expressed as means ± standard error, significance was observed at *P < 0.05 and **P < 0.01.

The activities of the antioxidant enzymes (CAT, SOD, and GSH-Px) and contents of the oxidative product (MDA) in the gut tissues of the goat kids are summarized in Figure 1. In the jejunum tissues, the activities of GSH-Px were significantly higher (*P* < 0.05) in the control group compared with that in the diarrhea group, while there was no significant difference in the SOD and CAT activity observed between the control group and diarrhea group (*P* > 0.05). And the level of MDA was higher (*P* < 0.05) in the jejunum tissues and significantly higher (*P* < 0.01) in the ileum tissues from the diarrhea group compared with the control group. As a result, the levels of the antioxidants significantly decreased in the diarrhea goats compared with the control goats. In contrast, the MDA levels in the gut tissues were significantly greater in the diarrhea goats than in the healthy control goats.

### The Level of IL-2 and IL-6 in the Intestinal Tissues

**Figure 2:**
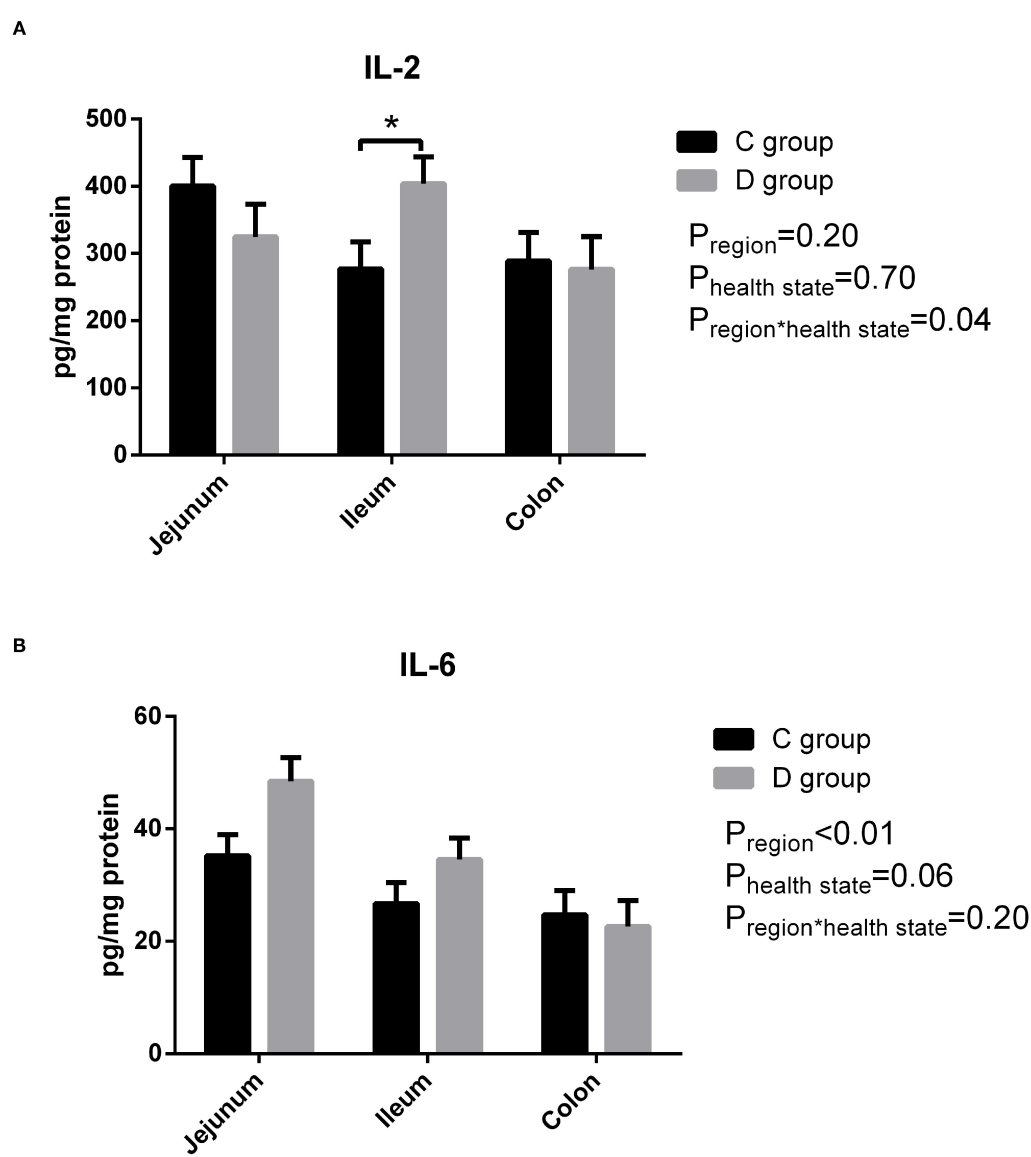
The level of intestinal IL-2 **(A)** and IL-6 **(B)** in neonatal diarrhea goats. C group means control group; D group means diarrhea group. Results are expressed as means ± standard error, significance was observed at *P < 0.05 and **P < 0.01.

As shown in Figure 2, there was a significant difference in that the level of IL-2 was significantly higher only in the ileum homogenate in the diarrhea group compared to the control group, and there was no significant difference in IL-6 observed between the control group and diarrhea group (*P* > 0.05). In short, there was no significant change in the levels of pro-inflammatory cytokines IL-2 and IL-6 in diarrhea goats compared with the control goats.

### Jejunum Transcriptome

**Figure 3:**
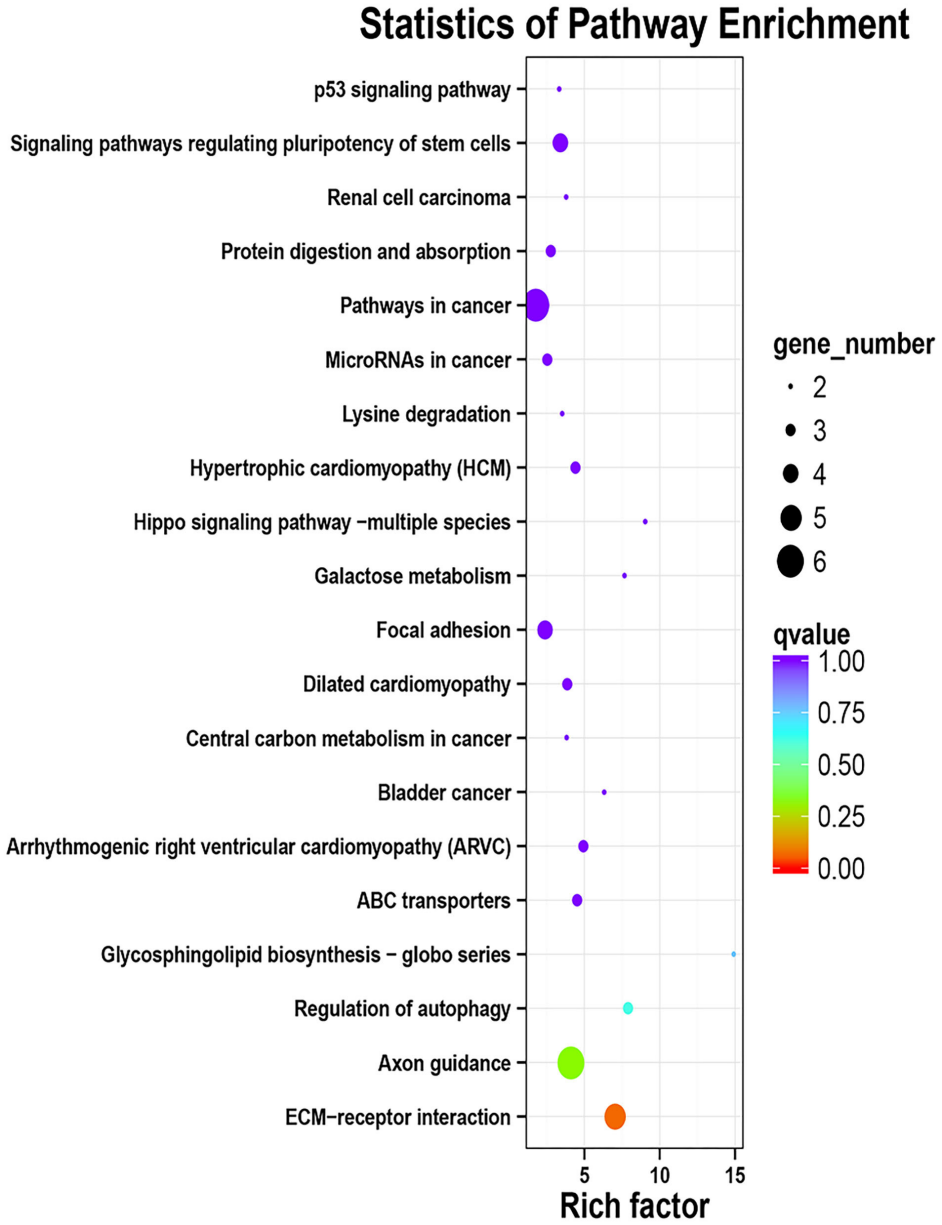
KEGG enrichment analyses of the up-regulated DEGs in jejunum of neonatal goats suffering from diarrhea as compared with control goat kids. The vertical axis represents the pathway category, and the horizontal axis represents Enrichment Factor which means the proportion of DEGs annotated to the pathway on genes annotated to the pathway.

**Figure 4:**
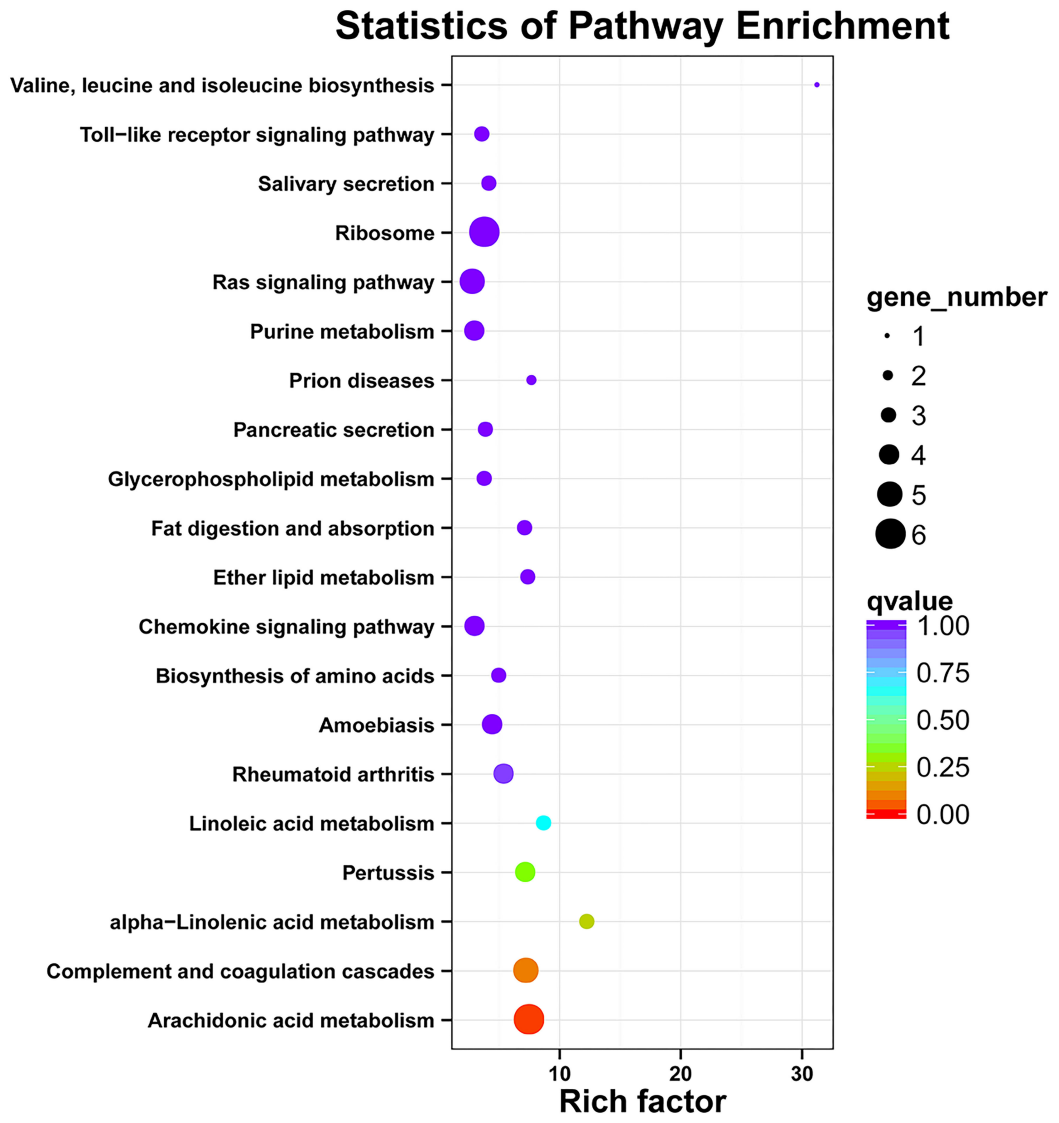
KEGG enrichment analyses of the down-regulated DEGs in jejunum of neonatal goats suffering from diarrhea as compared with control goat kids. The vertical axis represents the pathway category, and the horizontal axis represents Enrichment Factor which means the proportion of DEGs annotated to the pathway on genes annotated to the pathway.

A total of 163 million high-quality 100-bp paired-end reads (clean reads) were obtained from all of the samples (Supplementary Table 2), and among all of the clean reads, 95.82–96.41% were mapped for each sample (Supplementary Table 3). Moreover, there was a high mapping rate with uniquely mapped reads; a range of 87.82–91.13% of reads aligned to the reference genome. The principal component analysis of the transcriptome profiles indicated that the healthy control and diarrhea groups do look transcriptionally similar (Supplementary Figure 1). When the DEG between the diseased goats and healthy goats was further explored (the FDR < 0.05 and |log_2_ (foldchange) | ≥ 1), a total of 364 DEGs showed a different expression, in which 197 genes had a higher expression in the diarrhea goats than the control goats, whereas 167 genes had a lower expression in the diarrhea goats than in the control goats (Supplementary Table 4). The top GO analyses showed DEGs were mainly involved in the zinc ion binding (22 DEGs) and nucleoplasm (40 DEGs), only 19 DEGs involved in the inflammatory response (Supplementary Figure 2; Supplementary Table 5). The KEGG functional enrichment analysis of up-regulated DEGs identified pathways modified by the diarrhea, mainly related to the ECM-receptor interaction and Axon guidance. Down-regulated DEGs were mainly related to the Arachidonic acid metabolism, complement and coagulation cascades, and alpha-Linolenic acid metabolism. The top 20 KEGG enrichment pathways of up-regulated and down-regulated DEGs in jejunum of diarrhea kids are shown in Figures 3, 4, respectively.

### Expression of the Genes Related to the TLR4 Signaling Pathway and Barrier Function

RT-PCR was used to analyze the expression of the genes related to the intestinal barrier function and genes related to the TLR4 signaling pathway-related molecules. The results showed that the genes related to the intestinal TLR4 pathway apart from *MyD88*, such as *TLR4, TRAF6, IFN-*β, *IL-1*β, *TNF-*α, *IL-6, NLRP3, IRF3, TBK1*, and *NF-*κ*B p65* (Table 2), and genes related to the intestinal barrier function apart from *MUC20* and *ZO2*, such as *MUC12, MUC13, Occludin, CLDN1*, and *CLDN4* (Table 3), showed no significant difference between the control group and diseased group (*P* > 0.05). The expression levels of *MUC20* in the colon and *ZO2* in the ileum were significantly higher in the control group compared with the diarrhea group, while the expression of *MyD88* in the jejunum was significantly higher in the diarrhea group compared to the control group. These results indicated that there was no extraordinary difference between the healthy control and diarrhea groups of goats in the expression of most of the TLR4 signaling pathway-related genes and barrier function genes.

**Table 2:** The expression of genes related to TLR4 pathway in the lower gut.

**Item**	**Jejunum**		**Ileum**		**Colon**			***P*****-value**		
	**C**	**D**	**C**	**D**	**C**	**D**	**SEM**	**Gut**	**Treat**	**Interaction**
MyD88	1.96^a^	0.17^b^	1.25^b^	2.06^a^	0.20	0.45	0.31	<0.01	0.18	<0.01
TLR4	1.43	3.06	1.05	1.72	1.43	0.70	1.04	0.26	0.39	0.28
TRAF6	0.97	1.04	1.03	1.15	1.40	1.61	0.52	0.35	0.67	0.98
IFN-β	2.16	4.59	1.38	1.88	0.63	0.96	1.74	0.11	0.29	0.64
IL-1β	2.27	4.24	1.37	1.74	0.74	0.45	1.50	0.05	0.43	0.55
TNF-α	0.64	0.94	1.08	1.15	0.49	0.36	0.24	0.00	0.58	0.45
IL-6	0.27	0.45	1.31	0.63	4.80	2.78	1.46	0.00	0.32	0.56
NLRP3	0.53	0.61	1.77	1.63	0.49	1.16	0.42	0.00	0.40	0.37
IRF3	0.90	1.22	1.07	1.12	1.24	1.34	0.31	0.56	0.39	0.81
TBK1	0.85	1.09	1.05	0.84	0.47	0.76	0.23	0.23	0.42	0.27
NF-κB p65	0.73	0.71	1.05	1.21	0.64	0.63	0.19	0.00	0.68	0.74

a,b *Means in the same row with different superscript letter are significantly different (P < 0.05)*.

*C means control group; D means diarrhea group; MyD88, myeloid differentiation factor 88; TLR4, Toll-like receptor 4; TRAF6, TNF receptor-associated factor-6; IFN-β, interferon -β; IL-1β, Interleukin-1 beta; TNF-α, TUMOR necrosis factor-α; IL-6, Interleukin-6; NLRP3, pyrin domain-containing 3; IRF3, Interferon regulatory factor 3; TBK1, TANK-binding kinase-1; NF-κB p65, NF kappa B p65; CONT, control group; DISE, disease group*.

**Table 3:** The expression of immune genes in the lower gut.

**Item**	**Jejunum**		**Ileum**		**Colon**			***P*****-value**		
	**C**	**D**	**C**	**D**	**C**	**D**	**SEM**	**Gut**	**Treat**	**Interaction**
MUC12	2.66	1.41	3.53	1.91	2.20	2.06	1.03	0.64	0.13	0.61
MUC13	1.26	1.02	1.06	0.82	0.67	0.97	0.32	0.39	0.77	0.44
MUC20	1.21	2.38	0.87	1.61	7.76^a^	5.45^b^	1.01	<0.01	0.83	0.06
Occludin	1.07	1.35	1.08	1.21	0.79	0.79	0.24	0.07	0.36	0.75
ZO2	1.06	1.27	1.69^a^	1.04^b^	1.24	1.43	0.33	0.66	0.68	0.14
CLDN4	1.33	1.50	1.42	2.16	1.33	1.49	0.64	0.67	0.37	0.79
CLDN1	1.74	2.49	1.74	2.23	4.47	3.63	1.48	0.12	0.88	0.74

a,b *Means in the same row with different superscript letter are significantly different (P < 0.05)*.

*C means control group; D means diarrhea group; MUC12, Mucin12; MUC13, Mucin13; MUC20, Mucin20; TLR3, Toll-like receptor 3; ZO2, Zona Occludens 2; CLDN4, Claudin 4; CLDN1, Claudin 1; CONT, control group; DISE, disease group*.

## Discussion

Diarrhea is the most common disease in the newborn stage and infectious causes such as bacteria, viruses and protozoa are usually blamed (24). Wet weather in spring and summer and immature immune function were associated with increased neonatal diarrhea. Yatoo et al. (25) have reported neonatal diarrhea usually occurs during spring season from March to May and was corroboration with Parray et al. (26). Mortality rates of neonatal diarrhea depended on management and epidemic prevention conditions and varied 25–50% (27) and was 14.3% in the present study carried out from March to July in a small local family farm. And in this study, we showed that neonate goats with diarrhea exhibited an oxidative stress status in the intestine as evidenced by an increase in the MDA level and a decrease in GSH-Px level. Aslan et al. (28) reported MDA levels were found significantly higher whilst the GSH-Px, CAT, and SOD activities were significantly lower in lambs with Pestivirus than that of controls and trinitrobenzenesulfonic acid (TNBS)-induced colitis in mice, which was widely used as Crohn's disease (CD) models, showed the same result—that the enzyme activity of GSH-Px was significantly decreased, while MDA was significantly increased in the TNBS group compared with the blank group (29). Our results which are in accordance with the previous studies demonstrating damaged tissues undergo more free radical reactions than healthy ones (28–30) and intestine as the main site for the presence of a large number of microorganisms, nutrients, and interactions between immune cells, was very susceptible to peroxidation (31). The reason may be that the invasion of gastrointestinal pathogens are potent oxidizing stimuli which induces immune reactions to cope with the attack of pathogens by activating the activity of neutrophils and macrophages, resulting in excessive ROS production and accumulation, eventually resulting in oxidative stress (32). GSH-Px helps strengthen the oxidative defense system by catalyzing the reduction of harmful peroxides into harmless compounds and protecting the cell membrane structure and function (33). The increased level of MDA suggested an enhanced peroxidation of the membrane lipids under the attack of ROS, is a typical marker for the degree of oxidative stress and cell injury (34). From this study, both GSH-Px and MDA may be used as biomarkers for neonatal diarrhea.

Numerous studies have reported that animals had a significant increase in their immune response for the increased production of proinflammatory cytokines and decreased releasing of anti-inflammatory cytokines in response to infection (35, 36). However, the results from our study showed that there was no significant difference in the secretion of pro-inflammatory cytokines in the diarrhea goats compared with healthy control goats, both for IL-2 and IL-6, although the level of IL-2 was significantly higher in the ileum homogenate in the diarrhea goats. In contrast, Shi et al. (37) reported that the levels of IL-6 and IL-2 in the gut were increased in piglets orally infected with *C. perfringens* type C. In addition, IL-6 proved to be a useful prognostic marker in neonatal calf diarrhea (38). Then the expression of the entire genome of the jejunum transcriptome was determined under the circumstances and the PCA analyses of transcriptome data in jejunum showed no clear separation between the healthy group and diarrhea group, and the top GO analysis showed that DEGs were mainly involved in zinc ion binding and nucleoplasm and the KEGG pathway analysis proved DEGs were not related to immunity. These results indicate there being little biological difference in diarrhea and healthy control goats but as the sample size of jejunum transcription is very small it is difficult to be conclusive. It is clear that TLR4 and its downstream signaling pathways play a pivotal role for inducing the secretion of inflammatory cytokines during bacterial infection and diarrhea involve profound alterations in the mechanism controlling gut barrier function which may increase the intestinal permeability to pathogenic bacteria (37–39), aroused our attention. The real-time PCR results of various gut regions showed that the majority of the TLR4 pathway-related genes have no significant differences between the two groups apart from MyD88, which mainly mediates the production of pro-inflammatory cytokines by activating a series of toll-like receptor signaling pathways (40), was significantly higher in the ileum of the diarrhea goats. The expression of genes related to the intestinal barrier function apart from MUC20 and ZO2 was the same in both the diarrhea and control goats. Mucins form the first line of innate immunity *in vivo* and MUC20 is a part of the membrane-bound mucins and are highly expressed in the colon (41). Intercellular tight junctions (TJs) are closely related to the integrity of the intestinal barrier, and ZO2 as a scaffolding protein was studied. It was reported that the expression of MUC20 was down-regulated in ulcerative colitis mucosa (42), and our results showed that the expression of MUC20 in the colon and ZO2 in the ileum were significantly lower in the diarrhea goats, which was consistent with the previous study. Therefore, we speculate that neonatal goats suffering from diarrhea in this study mostly be infectious diarrhea caused by insufficient immunity. Our PCR data also provides plausible evidence that there was no strong inflammatory response occurring in the diarrhea goats.

Numerous studies have shown that intestinal inflammation was coupled to the increase of oxidative stress (43), but in our study, diarrhea goats experienced more free radical reactions compared with healthy goats but the change of the immune function was not obvious. The difference may have occurred for several reasons: (a) the overwhelming production of ROS in the intestine may have occurred before the immune cells reached the intestinal mucosa (44) and the “free radical induction theory” suggests the inflammation in intestinal mucosa is triggered by oxidative stress (45) due to the fact that oxidants produced by oxidative stress are activators of NF-κB, a crucial regulator for the activation of inflammation (46). In addition, ROS is involved in intermicrobial competition (47) and was proved by a recent study in which the increased concentration of ROS in the intestine accompanied by an expansion of the *E. coli* population in weaned piglets (48), suggesting an important role of the oxidative stress for the onset of infectious diseases; (b) it was reported that *Salmonella enterica* serovar Enteritidis and *S. Typhimurium* caused a strong inflammatory response while *S. Pullorum* induced the systemic infection of chicks without obvious inflammation (49), and the occurrence of inflammation is probably related to the invading pathogenic microorganisms; (c) the observable differences could relate to the fact the animals are naturally infected and the degree of variation between individuals in “diarrhea” status.

In conclusion, our data revealed that the predisposition to diarrhea is closely associated with intestinal oxidative stress in neonatal goats while there was no significant difference in the intestinal inflammatory status. This study revealed oxidative stress may be the potential mechanism underlying the pathophysiology of diarrhea in newborn goats, which suggests that the antioxidative mechanisms in the intestine may be more important for health than we previously appreciated. The antioxidant activity could act as an indicator of health status. The biomarkers of oxidative stress such as GSH-Px and MDA might be used to reflect the mortality and morbidity for young goats. This study has novelty in generating baseline information pertaining to changes associated with neonatal diarrhea in kids that can have both physio-biological- and production- related importance. And the current study also has several limitations. The insufficient sample size for the experiment was conducted in a small-scale goat farm and should be greater in future studies. Especially, the insufficient sample size of transcriptome analysis due to funding constraints. In addition, no etiopathogenic diagnosis was made in this study based on an epidemiological perspective and nasal discharge and loose stool could be bacterial or viral or environmental or food related. The immune response will vary based on the stressor listed here. Further study with an accurate diagnosis of the cause of diarrhea in neonatal goats, expansion sample size, and combine multi-omics analysis is warranted to further screen the biomarkers of diarrhea.

## Conclusion

Neonatal goats suffering from diarrhea exhibited strong oxidative stress in gut. Whereas no significant changes of proinflammatory cytokines secretion and immune-related genes expression were observed in diarrhea goat. In conclusion, oxidative stress may be the prominent mechanism underlying the pathophysiology of diarrhea newborn goats.

## Data Availability Statement

The jejunum RNA-seq data from this study have been submitted to the Sequence Read Archive (SRA) database (https://submit.ncbi.nlm.nih.gov/about/sra/) and the data are accessible through SRA Series accession number PRJNA635255 (https://www.ncbi.nlm.nih.gov/bioproject/PRJNA635255). Other data including activities of antioxidant enzymes, contents of oxidative product and pro-inflammatory cytokines and relative gene expression generated during this study are included in this published article.

## Ethics Statement

The animal study was reviewed and approved by the Institutional Animal Care and the Use Committee of the Institute of Subtropical Agriculture, Chinese Academy of Sciences, Changsha, China.

## Author Contributions

YC, ZH, and ZT contributed to experimental design. ZH, YC, and CY conducted the animal and laboratory experiments, collected samples. YC and ZH prepared libraries, acquired and analyzed the data, interpreted the results, and draft the writing. ZT and ZH revised the manuscript. All authors read and approved the final version of the manuscript and approved publication.

## Conflict of Interest

The authors declare that the research was conducted in the absence of any commercial or financial relationships that could be construed as a potential conflict of interest.

## Supplementary Material

Supplementary Figure 1 The principal component analysis of the transcriptome profiles in jejunum of neonatal goats suffering from diarrhea as compared with control goats. CTRL means control group; DIS means diarrhea group.

Supplementary Figure 2 The GO analyses of DEGs in jejunum of neonatal goats suffering from diarrhea as compared with control goats.

Supplementary Table 1 The primers of genes in this study.

Supplementary Table 2 Sequencing results of all samples.

Supplementary Table 3 Percentage of reads mapped to reference genome of all samples.

Supplementary Table 4 List of differential expression genes in neonatal diarrhea goats compare with control healthy goats.

Supplementary Table 5 List of top GO terms. Annotated means the number of Genes annotated to the GO term; Significant means the number of DEGs annotated to the GO term; Expected means the expected value of the number of DEGs annotated to the GO term; KS means the statistical significance of the GO term [the larger the –log10 (KS) value, the more significant the enrichment].

## Abbreviations

ROS: reactive oxygen
CAT: catalase
SOD: superoxide dismutase
GSH-Px: glutathione peroxidase
MDA: malondialdehyde
IL-2: interleukin-2
IL-6: interleukin-6
TLR4: Toll-like receptor 4
MyD88: myeloid differentiation primary response 88
TRAF6: TNF receptor associated factor 6
IFN-β: interferon-beta
IL-1β: interleukin 1beta
TNF-α: tumor necrosis factor alpha
NLRP3: leucine Rich Repeat And Pyrin Domain Containing 3
IRF3: interferon regulatory factor 3
TBK1: TANK-binding kinase 1
NF-κB p65: nuclear factor kappa-light-chain-enhancer of activated B-p65
RT-PCR: reverse transcription quantitative real-time PCR
TJs: tight junctions
CLDN1: Claudin1
CLDN4: Claudin4
ZO2: Zona Occludens2
MUC12: Mucin12
MUC13: Mucin13
MUC20: Mucin20
DEG: differential expression gene.
