# Supplementary figures and images for: Changes of Intestinal Oxidative Stress, Inflammation, and Gene Expression in Neonatal Diarrhoea Kids
Source: Front Vet Sci. 2021 Feb 4;8:598691. doi: 10.3389/fvets.2021.598691 (PMC7890263; doi:10.3389/fvets.2021.598691)

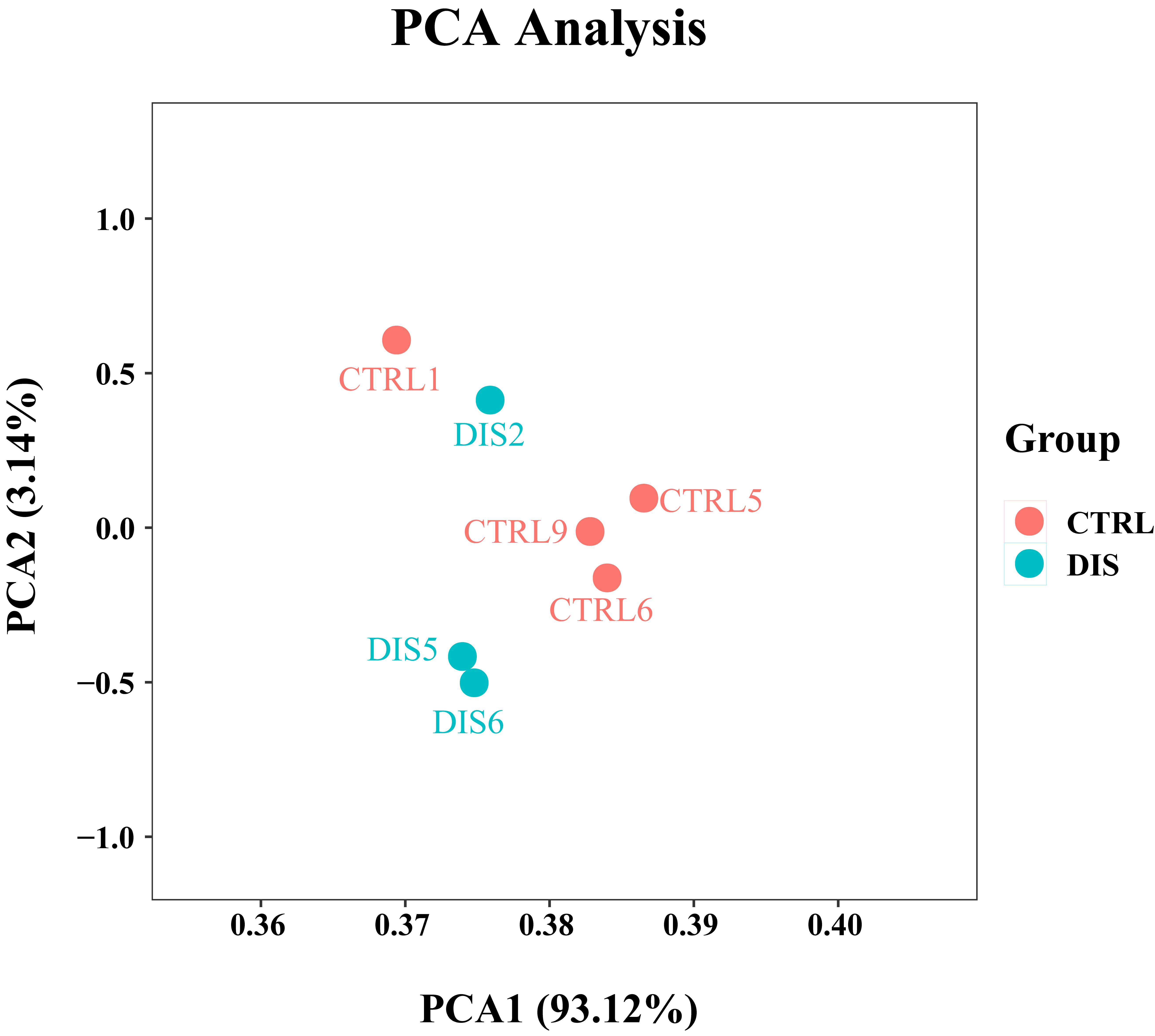

Supplement: Supplementary Figure 1 — The principal component analysis of the transcriptome profiles in jejunum of neonatal goats suffering from diarrhea as compared with control goats. CTRL means control group; DIS means diarrhea group. [file Image_1.tif]

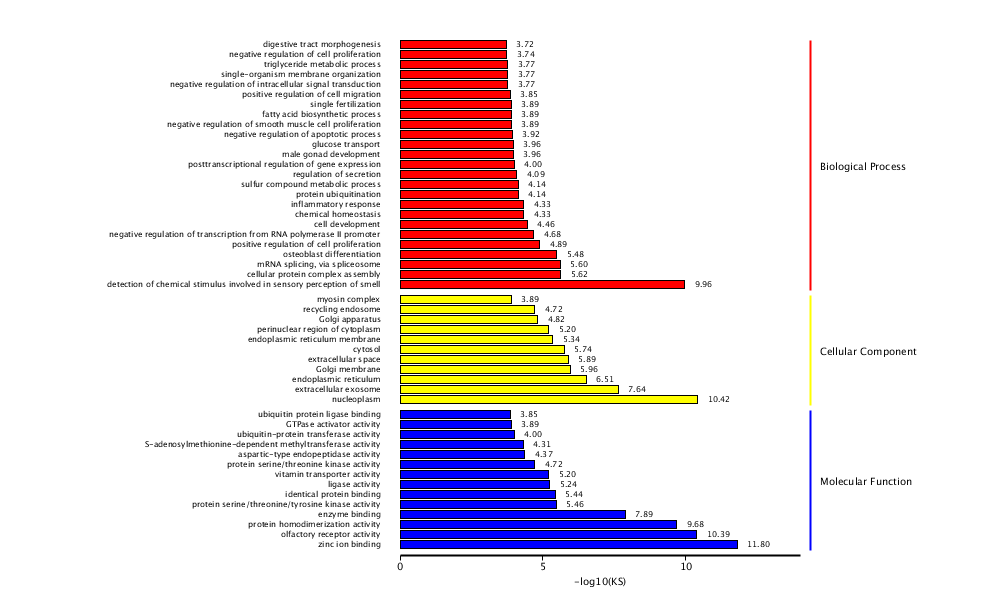

Supplement: Supplementary Figure 2 — The GO analyses of DEGs in jejunum of neonatal goats suffering from diarrhea as compared with control goats. [file Image_2.png]
