# Supplementary material for: Changes of Intestinal Oxidative Stress, Inflammation, and Gene Expression in Neonatal Diarrhoea Kids
Source: Front Vet Sci. 2021 Feb 4;8:598691. doi: 10.3389/fvets.2021.598691 (PMC7890263; doi:10.3389/fvets.2021.598691)
Supplement: Supplementary Table 1 — The primers of genes in this study. [file Data_Sheet_1.docx]

**Table S1.** The primers of genes in this study.

| Gene Name | Accession number | | | Primer sequences (5' to 3') | | Product size | |
| --- | --- | --- | --- | --- | --- | --- | --- |
| GAPDH | XM_005680968.3 | | | F：GGGTCATCATCTCTGCACCT  R：GGTCATAAGTCCCTCCACGA | | 176bp | |
| MyD88 | JQ308783.1 | | | F：TGCCTTCATCTGCTACT  R：GAGACAACCACCACCAT | | 168bp | |
| TLR4 | NM_001285574.1 | | | F：GTCAAGGACCAGAGGCA  R：GCTCATCTGACAAGTGGC | | 114bp | |
| TRAF6 | JQ308791.1 | | | F：CCGTGCACATTCAGTGCTTT  R：TGCGACTGGGTGTTCTCTTG | | 86bp | |
| IFN-β | JX458085.1 | | | F：GGAAGATGCCGTATTGGT  R：TTCCTTCTGGATTGGCTC | | 157bp | |
| IL-1β | DQ837160.1 | | | F：AAGGCTCTCCACCTCCTCTC  R：TTGTCCCTGATACCCAAGG | | 122bp | |
| TNF-α | NM_001286442.1 | | | F：CCACTGACGGGCTTTACCT  R：TGATGGCAGAGAGGATGTTG | | 141bp | |
| IL-6 | NM_001285640.1 | | | F：TGACTTCTGCTTTCCCTACCC  R：GCCAGTGTCTCCTTGCTGTT | | 193bp | |
| NLRP3 | XM_005682796.3 | | | F: GTCCGTTTCCTCTTTGG | | 148bp | |
|  |  |  |  | R: TGGGCTCAGTCTGTAGTGT | |  |  |
| IRF3 | | JQ308793.1 | F: AAGTGTTGCGTTTAGCGG | | 168bp | |  |
|  |  |  | R: GCACAATGTCTTCCTGGGT | |  |  |  |
| TBK1 | | KU182750.1 | F: AGACATACGCACCAAGC | | 105bp | |  |
|  |  |  | R: TCGGCAAGTAATCCACC | |  |  |  |
| NF-κB p65 | | IQ342088.1 | F: CAGCTCACAGATCGGGAAAAG | | 115bp | |  |
|  |  |  | R: CGGTGCTGTCTGGAAGGAA | |  |  |  |

**Table S2.** Sequencing results of all samples.

| Gene Name | | Accession number | | Primer sequences (5' to 3') | Product size |
| --- | --- | --- | --- | --- | --- |
| GAPDH | | XM_005680968.3 | F：GGGTCATCATCTCTGCACCT  R：GGTCATAAGTCCCTCCACGA | | 176 bp |
| MUC13 | | XM_005675237.3 | F：TGAGAAACGAGAGTGCTGCC  R：GCATGCCAAGAATGAGGACG | | 168 bp |
| MUC20 | | XM_005675240.3 | F：GAGAGTGGAGGAGAGCGAGAG  R：CACAAGAAACTAGACCCTCCCTAAT | | 180 bp |
| MUC12 | | XM_013976790.2 | F：TTTCCTGCATCCCACTTCGAG  R：TAGGGAGGCAGACGGAAACT | | 145 bp |
| TLR3 | XM_018041934.1 | | F：CAGCTGTTGTCACAGGCAAAA  R：GCAATCGGCTGCTTCATGTC | | 137 bp |
| Occludin | NC_030827.1 | | F：ATCGGAGTTTCAGGTGAATGGG  R：TCCGCCTGAAGAAGCAGAAAG | | 97 bp |
| CLDN4 | XM_005697785.2 | | F：CTGTTGGCAGGAAGGGAACT  R：ACGGCTACTTCACAGCAAGG | | 158 bp |
| ZO2 | XM_018052053.1 | | F：CTTGTGAGTGGGATTGGCATC  R：TCTGTTGGCACGTGTCTGTG | | 127 bp |
